# Remembering the bad times: Autobiographical memory fluency, specificity, and fading affect bias in adolescents at clinical risk of affective disorder

**DOI:** 10.1111/jcpp.70191

**Published:** 2026-06-17

**Authors:** Alice Roe, Siobhan Gormley, Eleanor Stephens, Eleanor Mapleston, Tim Dalgleish

**Affiliations:** ^1^ Medical Research Council Cognition and Brain Sciences Unit University of Cambridge Cambridge UK; ^2^ Cambridgeshire and Peterborough NHS Foundation Trust Fulbourn UK; ^3^ Department of Psychology University of Cambridge Cambridge UK

**Keywords:** Adolescence, affective disorders, memory, depression, anxiety

## Abstract

**Background:**

Distortions in autobiographical memory processing are implicated in the onset and maintenance of multiple mental health problems in adults. While reduced personal memory specificity has been identified as a transdiagnostic risk factor in adolescents, less is known about other domains of memory processing, including biases in the accessibility and affective intensity of negative and positive memories.

**Methods:**

We administered a novel recall paradigm, the Good Day‐Bad Day task (Hitchcock et al., *Journal of Experimental Psychology: General*, 2019, 149, 198), to assess autobiographical memory fluency for positive and negative events, memory specificity, and the Fading Affect Bias (FAB) – the extent to which the emotional intensity associated with positive memories fades less than for negative memories overtime – in adolescents, aged 16–18, at high (*n* = 201, *M*_age_ = 17.20, *SD* = 0.60, 84.6% female, 2% other) and low clinical risk (*n* = 117, *M*_age_ = 17.10, *SD* = 0.60, 77.8% female) of affective disorder.

**Results:**

We found that superior recall for positive relative to negative events and a strong FAB were associated with good mental health. Risk of affective disorder was associated with a significant reduction in these positive biases and recall of a higher number of negative memories compared with Low‐Risk adolescents. Memory specificity did not differ by risk status.

**Conclusions:**

Findings extend research on autobiographical memory processing to two novel domains in adolescence and further elucidate how differences in autobiographical memory recall may underlie mental health, with implications for the development of memory‐based interventions.

Retrieving and recollecting memories of past experiences is a fundamental part of daily life (Conway & Pleydell‐Pearce, 2000). From recalling specific memories of particular events (‘my 16th birthday’) to reflecting on generalised summaries of past events (‘my childhood birthday parties’) or extended periods of time (‘when I was at school’), autobiographical memory underpins our ability to predict and plan for future events, establish social connections, and make judgements about the self and the world (Dalgleish & Hitchcock, 2023). During adolescence, autobiographical memory is organised within a hierarchical ‘self‐memory system’, as discrete episodic memories are thematically integrated to establish a complex narrative identity (Fivush, 2011; Habermas & Bluck, 2000; Habermas & de Silveira, 2008). The organisation of autobiographical memory is fundamental to an emerging self‐concept and mental life in adolescence, with the potential to both support and undermine mental health (Conway & Pleydell‐Pearce, 2000). In adults, positive biases in memory processing are associated with good mental health (e.g. Hitchcock, Rees, & Dalgleish, 2017), while distortions in these processes are an established transdiagnostic feature of several mental health conditions (Dalgleish & Hitchcock, 2023). It is hypothesised that biases in autobiographical memory recall are maintained by a hierarchy of negative (or positive) self‐schemas that facilitate preferential access to schema‐congruent memories (Conway & Pleydell‐Pearce, 2000; see Dalgleish & Hitchcock, 2023 for a full account of this theoretical framing).

Adolescence is a crucial developmental period for the onset of several mood and anxiety‐related disorders, with early onset associated with worse outcomes (Solmi et al., 2022). Differences in autobiographical memory processes may represent a transdiagnostic risk factor for poor mental health in adolescence. To date, research with adolescents has focused on reduced memory specificity – a difficulty recalling memories for individual past events in preference for memories for extended periods of time or categories of events. Evidence from a longitudinal individual participant meta‐analysis demonstrated that reduced memory specificity is a transdiagnostic risk factor, predicting increased risk of psychiatric disorders in adolescence (Doan et al., 2025). Cross‐sectionally, reduced memory specificity has consistently been associated with depression symptoms (Hitchcock, Nixon, & Weber, 2014; Kuyken, Howell, & Dalgleish, 2006; Park, Goodyer, & Teasdale, 2002; Warne, Caseras, & Rice, 2020) and trauma exposure in adolescence (Hitchcock et al., 2014; Nixon, Ball, Sterk, Best, & Beatty, 2013; Valentino, Toth, & Cicchetti, 2009).

In adults, there is a large body of research demonstrating biases in other domains of autobiographical memory recall (see Dalgleish & Hitchcock, 2023 for a review). This includes biased recall of negative autobiographical memory content in adults with depression, anxiety, and PTSD. For example, depressed individuals recall more negative content (Gotlib & Joormann, 2010), show reduced category fluency for positive past events (Hitchcock et al., 2019), and access negative autobiographical memory content faster compared with nondepressed controls (Lloyd & Lishman, 1975). There is also evidence that negative memories maintain their affective intensity in depressed compared with healthy adults. The so‐called fading affect bias (FAB) is a well‐established phenomenon whereby, for healthy individuals, the emotional intensity associated with positive memories fades less than for negative memories over time – a process which is postulated to maintain good mental health (Walker, Skowronski, Gibbons, Vogl, & Thompson, 2003). Conversely, the FAB is reduced in adults with elevated anxiety (Walker, Yancu, & Skowronski, 2014) or depressive symptoms (Hitchcock et al., 2019).

Despite the apparent central role of these processes in the onset and maintenance of mental health problems, there has been less research exploring these other domains of autobiographical memory processing in adolescents. Several cross‐sectional studies have demonstrated that adolescents with elevated depression and anxiety symptoms show a recall bias for negative relative to positive words (Bone, Lewis, Roiser, Blakemore, & Lewis, 2021; Fattahi Asl, Ghanizadeh, Mollazade, & Aflakseir, 2015; Neshat‐Doost, Taghavi, Moradi, Yule, & Dalgleish, 1998; Platt, Waters, Schulte‐Koerne, Engelmann, & Salemink, 2017; Watts & Weems, 2006). However, there is limited research on biased recall of autobiographical material specifically. One study reported that, following a negative mood induction, adolescents with current or remitted depression had difficulty recalling rich positive memories compared with healthy controls (Begovic et al., 2017). Kuyken and Howell (2006) also found that depressed adolescents preferentially rehearsed negative memories compared with never‐depressed adolescents. It is also not understood whether the FAB is present in adolescents, or its association with mental health. One study did find evidence for a FAB among children aged 8–12 for events occurring in the past year (Rollins, Gibbons, & Cloude, 2018). However, there has been no research investigating the FAB in adolescents as a function of mental health.

## The present study

The aim of this study was therefore to examine multiple facets of autobiographical memory recall in older adolescents as a function of mental health risk. Specifically, we were interested in examining memory biases as a candidate transdiagnostic risk factor, given evidence of their transdiagnostic utility in adults (Dalgleish & Hitchcock, 2023) and emerging evidence in adolescents (Doan et al., 2025). Furthermore, recent evidence has shown that individuals at elevated risk typically experience comorbid and multiple diagnoses across the life course (Caspi et al., 2025), emphasising the need, particularly in adolescence, to identify profiles of risk associated with multiple mental health outcomes, beyond focusing on specific disorders. We focused on risk of affective disorder – specifically, depression, anxiety and posttraumatic stress disorder (PTSD) – given their high comorbidity and clustering within hierarchical taxonomies of mental health (Kotov et al., 2017).

To assess autobiographical memory recall, we used a novel paradigm, the Good‐Day Bad‐Day (GD‐BD) Task (Hitchcock et al., 2019), in which participants are instructed to recall as many ‘good‐day’ and ‘bad‐day’ memories within 2 min. They also provide a description of each event, which is coded for specificity, and rate the emotional intensity of memories at the time they occurred and in the present. Hitchcock et al. (2019) found superior fluency in generating good‐day relative to bad‐day memories in healthy adults, but no difference in depressed adults. Depressed adults also demonstrated reduced memory specificity of both good‐day and bad‐day memories, and a reduced FAB. We aimed to examine autobiographical memory fluency for positive and negative events and the FAB in adolescents for the first time.

A secondary aim was to further examine memory specificity. Most research examining autobiographical memory specificity in adolescents has used the Autobiographical Memory Test (AMT; Williams et al., 2007). In the AMT, participants are asked to generate a specific memory in response to individual cue words, usually of positive or negative valence. While a robust finding, it is possible that the association between reduced memory specificity and mental health may be an artefact of the AMT in adolescents. The use of individual cues makes the AMT cognitively demanding and assesses somewhat artificial memory recall (Gutenbrunner, Salmon, & Jose, 2019; Salmon et al., 2021). By using broad category cues as opposed to individual cues, the GD‐BD task allows examination of memory specificity with minimal constraints.

We hypothesised that:

1. Adolescents at low risk of affective disorders would recall a greater number of positive compared with negative memories and this difference would be reduced among those at risk.
2. At‐risk adolescents would recall fewer specific memories compared with those at low risk.
3. The FAB would be reduced among at‐risk adolescents.

## Method

Methods, hypotheses, and statistical analyses were preregistered, as part of a larger study: https://osf.io/c48fn/overview. Ethical approval was granted by Cambridge Psychology Research Ethics Committee (PRE.2023.050).

### Participants

Participants, aged 16–18, were recruited from schools and sixth‐form colleges (taking students aged 16–18) around Cambridgeshire, UK. Participants were provided with information about the study at school/college (i.e., that it was a study investigating mental health in adolescence) and invited to sign‐up to a testing session. In total, 366 participants took part in the study between January–November 2024 (80.6% female; mean age: 16.64 years, *SD* = 0.64). There were no formal exclusion criteria, however, participants with self‐declared diagnoses of autism, ADHD and/or severe dyslexia were asked to inform researchers of this prior to the testing session and were set aside from analyses (*n* = 20). Following data quality checks (see below), a further 26 participants were removed. One participant did not recall any ‘bad‐day’ memories and was set aside from analyses, leaving a final sample of 318 for the main analyses.

### Materials and measures

#### The Good Day‐Bad Day Task

The GD‐BD task was adapted from Hitchcock et al. (2019) for computer‐based testing. First, participants were given 2 min each to recall as many specific good events (good‐days) and specific bad‐events (bad‐days) from their life as possible. Participants were told that they should only remember experiences from their past that were specific (i.e., ‘that happened in a particular instance or lasted for a day or less’). Participants were told that the events could have happened recently or a long time ago, and could be trivial or important, but must be from their own life. During the timed component, participants were instructed to write down one or two keywords that would remind them of the event later on. Comprehension was checked with a multiple‐choice question that had to be answered correctly before proceeding. The order of the two conditions (good‐days and bad‐days) was counterbalanced across participants. Next, participants were shown their keywords and asked to write a brief description of each memory (good‐day followed by bad‐day). This was self‐paced. In the final part of the task, participants were asked to rate the retrieved memories in terms of how happy/unhappy they had felt at the time the events occurred and also how happy/unhappy they feel in the present when reflecting back on the events, on a 7‐point Likert scale (extremely unhappy to extremely happy), and to state, as accurately as possible, when each event had occurred.

Three variables were computed from the task performance.

*Memory category fluency*. The total number of ‘good‐day’ and ‘bad‐day’ memories, respectively.

*Memory specificity*. Each memory description was coded as either ‘specific’ (events that happened in a particular instance and/or lasted for a day or less), ‘categoric’ (events that occurred repeatedly, e.g. Christmas), ‘extended’ (events that lasted for longer than a day, e.g. going on holiday), ‘associate’ (not a memory, e.g. reference to a future event, an opinion), or ‘omission’ if no memory was retrieved or a memory was repeated (Williams & Broadbent, 1986).

A natural language model developed for the purposes of scoring written autobiographical memories (Mistica et al., 2024) was used to score the memories in the present study. Initial comparison of scoring between the model and the ‘ground truth’ scores of the first author (*n* = 995 memories, 21.3%) identified some inaccuracies in the model (agreement rate of 84.0%). To improve accuracy, a screening method was developed whereby, following initial model scoring, a subset of memories was manually checked, based on both keyword searches relating to common themes or events that had been found to be associated with inaccurate scores by the model (e.g. bereavement) and on the model's own ‘precision’ score, indicating confidence in its output for each memory (<0.9). Full details of the approach used, including keywords, are available in the Supporting Information (see Appendix S1 and Table S1). This approach was tested on a further 758 memories (16.2%) and resulted in an improved agreement rate of 99.1%.

*Fading affect bias (FAB)*. The FAB index was calculated as in Hitchcock et al. (2019). Emotion ratings for the bad‐day memories were reverse scored so that the scales for the emotional intensity of good‐ and bad‐day memories were in the same direction. Mean emotional intensity ratings for ‘now’ were then subtracted from the emotional intensity ratings for ‘then’, separately for good‐day and bad‐day memories. The computed variable for good‐days was then subtracted from the computed variable for bad‐days to give a FAB score – the extent to which emotional intensity faded more for bad‐day memories relative to good‐day memories. A positive FAB score indicated that bad‐day memories faded more than good‐day memories.

#### Semantic category fluency

To measure category fluency for nonautobiographical material, participants completed a semantic category fluency task, in which they were instructed to write down as many animals as they could think of (trial one) and fruits or vegetables (trial two) within 1 min. These categories are commonly used in the literature (e.g. Arán Filippetti, Krumm, & López, 2023). Participants' scores were the mean number of correct responses across the two trials.

#### Mental health measures

The Centre for Epidemiological Studies Depression scale (CESD; Radloff, 1977) was used to measure depression symptoms. The CESD is a 20‐item self‐report scale which asks respondents to rate how often they have experienced symptoms associated with depression. The measure produces a total symptom scale and recommended cut‐offs to indicate elevated risk. In this study, we used a cut‐off ≥21, which is recommended as an indicator of ‘probable’ clinical caseness among community samples of adolescents (Cuijpers, Boluijt, & van Straten, 2008). The measure showed strong internal consistency, Cronbach's *α* = .91.

The Revised Child Anxiety and Depression Anxiety Subscale (RCADS; Ebesutani et al., 2012) was used to measure anxiety symptoms. The anxiety scale reflects a ‘broad anxiety’ dimension, comprising the various anxiety‐related conditions in the DSM (Generalised Anxiety Disorder, Obsessive Compulsive Disorder, Seasonal Affective Disorder, Specific Phobia, Panic Disorder). To calculate anxiety risk scores, total scores were converted to standardised *T*‐scores using established methods (https://rcads.ucla.edu/). A cut‐off score of ≥70 was used to indicate probable clinical caseness (Chorpita, Moffitt, & Gray, 2005). In this study, *α* = .86.

The Child and Adolescent Trauma Screen (CATS; Sachser et al., 2017) was used to measure posttraumatic stress symptoms (PTSS). First, participants indicated whether they had experienced 14 potentially traumatic events (e.g. car accident). Next, they completed a 20‐item questionnaire assessing PTSS. Participants were considered to be at risk of PTSD if they indicated exposure to a potentially traumatic event and scored above the recommended clinical cut‐off scores, indicating probable PTSD diagnosis (≥25; Sachser et al., 2022). In this study, *α* = .91.

#### Demographic information

Participants also provided their date of birth, ethnicity, and gender. Free school meal eligibility was used as a proxy for socioeconomic status (SES; Ilie et al., 2017).

### Procedure

Participants took part in an in‐person testing session at their school/college or at the MRC Cognition and Brain Sciences Unit. Testing sessions took place in small groups (*n* = 2–20) under exam‐style conditions. Participants completed a set of tasks and questionnaires lasting approximately 2.5 h, including additional measures not reported here (see preregistration). The GD‐BD task was always completed first, followed by the semantic category fluency task and questionnaires (demographic information, CESD, RCADS Anxiety Subscale, and CATS). For participants taking part in the study at school/college, the questionnaire component was completed without researcher supervision in the participants' own time within 7 days of the first session, to enable testing sessions to take place in the school day. The study was completed on laptops and data were collected via Qualtrics (www.qualtrics.com). Participants were compensated £30.

### Analysis

All analyses were preregistered: https://osf.io/c48fn. Any deviations from the preregistration are clearly marked in the text. Analyses were conducted in R v4.4.3 (R Core Team, 2025) using the *afex* (Singmann, Bolker, Westfall, Aust, & Ben‐Shachar, 2023) and *lme4* (Bates, Maechler, Bolker, & Walker, 2015) packages.

Preregistered multivariate outlier detection checks were conducted using Mahalanobis distance scores, compared with a chi‐squared distribution with the same degrees of freedom. Outliers with *p* < .001 were removed (*n* = 11). Additional data quality checks were undertaken (not preregistered) due to concerns raised during testing about participants for whom English was not a first language. Scores on the Mill Hill Vocabulary Scale, a measure of verbal intelligence which evaluates participants' understanding of 33 words, were inspected and participants scoring two standard deviations below the mean were excluded (scores ≤ 8). This was used as a proxy measure to identify participants that may have a poorer than average standard of English. This led to the removal of 15 participants. Analyses with outliers included are reported in the Appendix S2.

## Results

Participants were divided into an ‘At‐Risk’ or ‘Low‐Risk’ group depending on whether they scored above clinical cut‐offs on one or more of the CESD (≥21), CATS (≥25) and/or RCADS Anxiety subscale (≥70). Of the 318 participants, 201 were placed in the At‐Risk group. The majority of these participants scored above clinical thresholds in more than one measure, that is, were ‘transdiagnostically’ at risk (two measures: *n* = 74, 36.82%; three measures: *n* = 48, 23.88%).^1^ One‐hundred and seventeen participants scored below probable clinical thresholds on all three measures and were therefore placed in the ‘Low‐Risk’ group. Participant characteristics are presented in Table 1.

**Table 1:** Participant characteristics for the At‐Risk and Low‐Risk groups, mean (*SD*)

Variable	Total	Low‐Risk	At‐Risk
	*n* = 318	*n* = 117	*n* = 201
Age (years)	17.1 (0.6)	17.1 (0.6)	17.2 (0.6)
Gender [*n* (%)]			
Female	261 (82.1%)	91 (77.8%)	170 (84.6%)
Male	53 (16.7%)	26 (22.2%)	27 (13.4%)
Other	4 (1.3%)	0 (0%)	4 (2%)
SES [*n* (%)]			
High	258 (81.1%)	101 (86.3%)	157 (78.1%)
Low	60 (18.9%)	16 (13.7%)	44 (21.9%)
Depression (CESD)	22.7 (10.9)	12.6 (5.0)	28.5 (8.9)
Anxiety (RCADS)	60.5 (14.3)	49.7 (8.8)	66.7 (13.2)
PTSD (CATS)	22.9 (12.3)	12.3 (6.4)	29.2 (10.5)
Semantic category fluency	13.6 (3.4)	13.7 (3.7)	13.6 (3.2)

SES was calculated as low = eligible for free school meals, high = not eligible for free school meals. CATS, Child and Adolescent Trauma Screen; CESD, Center for Epidemiological Studies for Depression Scale; RCADS, Revised Child Anxiety and Depression Scale – Anxiety subscale.

The groups did not significantly differ in age, *t*(313) = 1.18, *p* = .24, *d* = 0.14, 95% CIs (0.09–0.37) or SES, *χ* ^2^(1) = 2.75, *p* = .10. Fisher's exact test revealed a near‐significant difference in gender, *p* = .051. When removing participants identifying as ‘Other’ (*n* = 4), this difference became less significant, *χ* ^2^(1) = 3.21, *p* = .07, although there was still a trend for more girls to be in the At‐Risk group than boys. Due to the small number of ‘Other’ participants, gender was not adjusted for in subsequent analyses. Demographic covariates were not correlated with the GD‐BD variables or mental health measures (all *r* < .14) and were not included in subsequent analyses.

There were expected between‐group differences in scores on the CESD, *t*(315.6) = 20.56, *p* < .001, *d* = 2.22, 95% CIs (1.49–2.49), RCADS, *t*(310.6) = 13.79, *p* < .001, *d* = 1.52, 95% CIs (1.27, 1.76), and CATS, *t*(315.4) = 17.76, *p* < .001, *d* = 1.94, 95% CIs (1.67, 2.20), with the At‐Risk group scoring higher in each case.

### GD‐BD task performance

Descriptive data for performance on the GD‐BD task are presented in Figure 1. A manipulation check verified that good‐day memories were rated more positively than bad‐day memories at the time of occurrence, *F*(1,316) = 15,794.69, *p* < .001, $n_{p}^{2}$=.98 [.98, .98]. There was no significant difference between risk group, *F*(1,316) = 2.80, *p* = .10, $n_{p}^{2}$=.01 [.00, .04], nor interaction between risk group and condition, *F*(1,316) = 0.001, *p* = .98, $n_{p}^{2}$=.00 [.00, .00], indicating that participants recalled memories of comparable affective intensity regardless of mental health status. In terms of when the memories occurred, there was a significant difference between good‐day and bad‐day memories, *F*(1,315) = 81.37, *p* < .001, $n_{p}^{2}$=.21 [.13, .28], with bad‐day memories taking place longer ago. There was no significant effect of risk group, *F*(1,315) = 1.05, *p* = .31, $n_{p}^{2}$=.01 [.00, .03], nor interaction between risk group and condition, *F*(1,315) = 2.44, *p* = .12, $n_{p}^{2}$=.01 [.00, .04].

**Figure 1:**
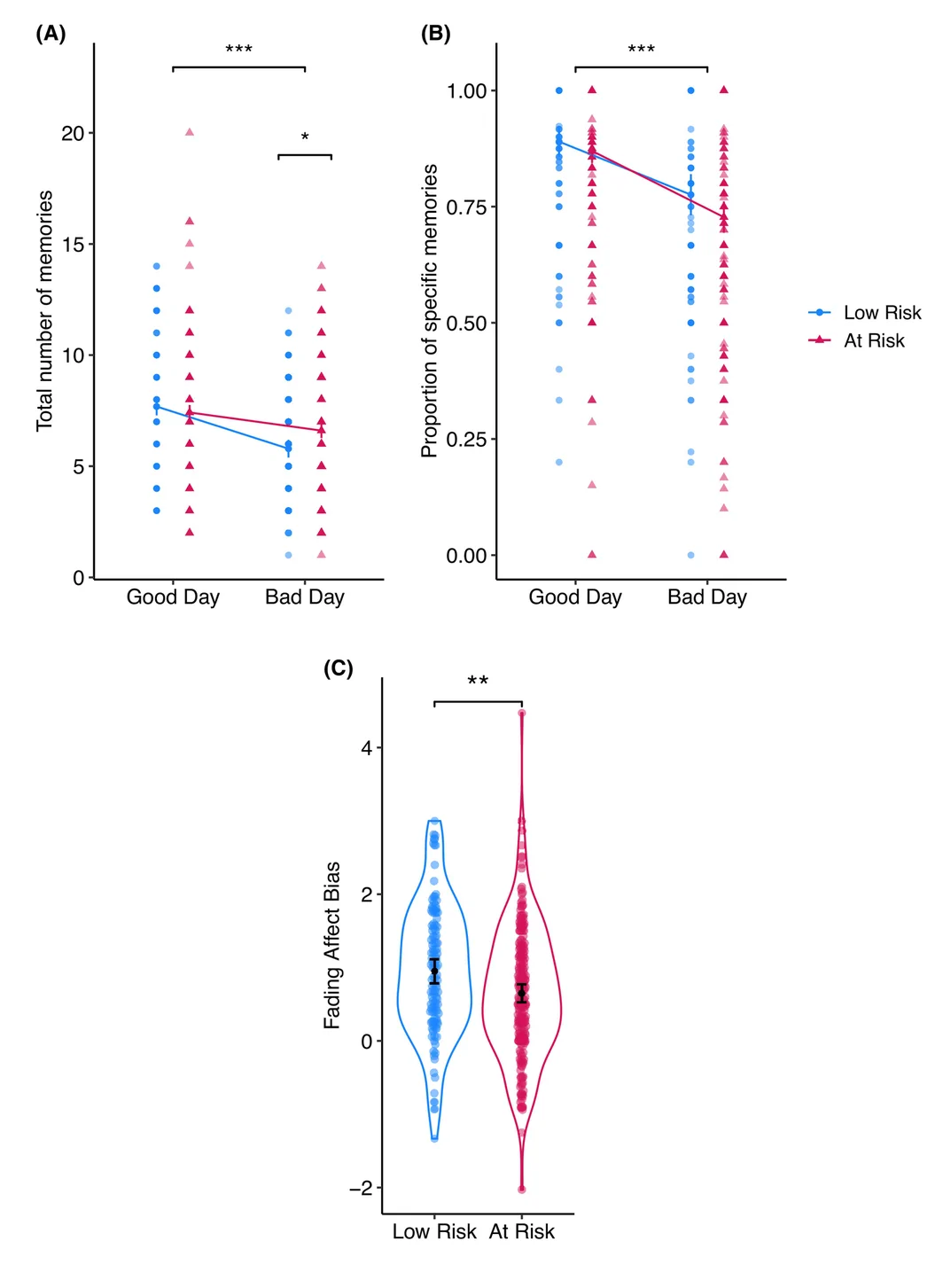
Performance on the GD‐BD task. (A) Total number of memories recalled in the good‐day and bad‐day conditions by risk group. Lines show group differences across the two conditions. Raw data points are shown, with darker gradient indicating overlapping points. (B) Proportion of recalled memories that were specific, by condition and risk group. (C) Fading Affect Bias index for the Low‐Risk and At‐Risk groups. Violin plots show data distribution. Error bars are 95% confidence intervals. **p < .01, ***p < .001

#### Memory category fluency

To compare differences between groups on the number of memories recalled in each condition, we conducted a repeated measures ANCOVA (co‐varying for scores on the semantic category fluency task as a measure of general fluency ability). Analyses were first performed with order (good‐days first vs. bad‐days first) included. There was no significant main effect of order or interaction with other variables (*F* < 1), hence analyses are presented without order as a factor.

There was a small but significant main effect of condition, *F*(1,312) = 4.81, *p* = .029, $n_{p}^{2}$=.02 [.00, .05], with a greater number of memories recalled in the good‐day condition, but no significant main effect of risk group, *F*(1,312) = 1.08, *p* = .30, $n_{p}^{2}$=.01 [.00, .03]. As expected, there was a significant interaction between condition and risk group, *F*(1,312) = 12.90, *p* < .001, $n_{p}^{2}$=.04 [.01, .09]. Post hoc comparisons showed that both groups recalled significantly more good‐day memories than bad‐day memories, but this difference was attenuated in the At‐Risk group in line with our hypotheses (At‐Risk: *b* = 0.82, *SE* = .18, *t*(314) = 4.45, *p* < .001; Low‐Risk: *b* = 1.90, *SE* = .24, *t*(314) = 7.93, *p* < .001). The At‐Risk group recalled significantly more bad‐day memories than the Low‐Risk group, *b* = 0.81, *SE* = .29, *t*(314) = 2.84, *p* = .01, providing support for our first hypothesis.

#### Memory specificity

To examine group differences in memory specificity, we calculated the proportion of specific memories recalled for each participant to adjust for any differences in the number of memories recalled by condition across groups. Our preregistration specified using a mixed ANCOVA with proportion of specific memories as the dependent variable; however, due to ceiling effects in the data (170 participants recalled only specific good‐day memories and 105 participants recalled only specific bad‐day memories) and violation of assumptions for linear regression, we instead used a generalised linear mixed effects model. Analyses were otherwise conducted exactly as prespecified and results were the same as the mixed ANCOVA (see Appendix S3). The final model included fixed effects of condition and risk group, their interaction, and random effects for participant. Results revealed a main effect of condition, such that good‐day memories were more likely to be specific, *b* = 1.07, *SE* = .15, *z* = 6.94, *p* < .001. Post hoc estimated marginal means revealed a predicted probability of a specific response of 0.92 (*SE* = .01, 95% CI [0.90, 0.93]) for good‐day memories, compared with 0.79 (*SE* = .02, 95% CI [0.76, 0.82]) for bad‐day memories. There was no significant main effect of risk group, *b* = −0.29, *SE* = .18, *z* = −1.63, *p* = .10, nor interaction between risk group and condition, *b* = .06, *SE* = .19, *z* = .31, *p* = .76. Our second hypothesis was therefore not supported.

In the literature on autobiographical memory specificity, a common analytic approach is to examine proportions of categorical, as opposed to specific, memories (e.g. Park et al., 2002). We therefore conducted an exploratory analysis (not preregistered) predicting the proportion of *categoric* memories. As above, the model included fixed effects of risk group and condition, their interaction, and random effects for participant. Results revealed that participants in the At‐Risk group recalled a lower proportion of categoric memories, but this difference did not reach statistical significance, *b* = −1.30, *SE* = .68, *z* = −1.91, *p* = .056. There was no significant main effect of condition, *b* = −.56, *SE* = .35, *z* = 1.62, *p* = .11. There was a significant interaction between risk group and condition, such that the difference between good‐day and bad‐day memories was larger for the At‐Risk group, compared with the Low‐Risk group, *b* = 1.34, *SE* = .49, *z* = 2.74, *p* = .006. Estimated marginal means indicated that, for bad‐day memories, At‐Risk participants recalled a lower proportion of categoric memories (predicted probability = .00018, *SE* = .00015, 95% CI [0.00003, 0.0009]) compared with Low‐Risk participants (predicted probability = .00065, *SE* = .00055, 95% CI [0.0001, 0.0034]). For good‐day memories, the estimated proportions were similar across groups (Low‐Risk: 0.00037, *SE* = .00032, 95% CI [0.000007, 0.00200]; At‐Risk: 0.00039, *SE* = .00031, 95% CI [0.00008, 0.00183]).

#### FAB

One‐sample *t*‐tests with a reference value of zero demonstrated a significant FAB for both groups: Low‐Risk, *M* = .95, *SD* = 0.90, *t*(116) = 11.44, *p* < .001, *d* = 1.06 [0.83, 1.28]; At‐Risk, *M* = .65, *SD* = 0.9, *t*(200) = 10.23, *p* < .001, *d* = 0.72 [0.57, 0.88]. A one‐way ANOVA revealed that the FAB index was significantly larger in the Low‐Risk group compared with the At‐Risk group, *F*(1,316) = 8.54, *p* = .004, $n_{p}^{2}$=.03 [.00, .07], providing support for our third hypothesis (see Figure 1).

## Discussion

This study utilised a novel paradigm, the GD‐BD task, to examine facets of autobiographical memory processing – category fluency, affective intensity, and memory specificity – as a function of mental health risk status in adolescents. In line with our first hypothesis, results revealed that both groups showed greater autobiographical memory fluency for positive (good‐days) compared with negative (bad‐days) memories, although this difference was attenuated in the At‐Risk group. This finding indicates that mental health in adolescence is associated with more fluent recall of associated sets of positive memories compared with negative memories.

Positive biases are posited to play a key role in maintaining mental health (Taylor & Brown, 1988) and previous research has shown that biases in self‐perception (Hards, Ellis, Fisk, & Reynolds, 2019; Orchard & Reynolds, 2018), future‐directed thinking (Uribe, Neira Espejo, & Pedroso, 2022), and interpretation of ambiguous events (Orchard & Reynolds, 2018; Parsons, Songco, Booth, & Fox, 2021) are well established by mid‐adolescence. Here, we extend this work to a novel cognitive domain – recollective fluency for positive autobiographical memories. Importantly, transdiagnostic mental health risk was associated with a relative reduction in the magnitude of this positive bias, rather than its absence or an overt negative bias. This suggests that mental health risk may be driven by a reduction in these positive self‐serving biases and the reduced accessibility of positive autobiographical content.

Second, we found evidence that the FAB was significantly attenuated among At‐Risk adolescents, although the effect was nevertheless present in both groups. Despite being a well‐evidence phenomenon in adults, there is minimal research on the development of the FAB in childhood and adolescence (Rollins et al., 2018). Our finding demonstrates that the FAB is well‐established by mid‐adolescence and supports mental health, perhaps indicative of the strength of positive biases in affect‐related processing that characterise human cognition (Diener, Oishi, & Lucas, 2003; Walker & Skowronski, 2009). As with memory category fluency, poor mental health was not characterised by the absence or reversal of this positive bias, but a relative reduction in its magnitude.

These findings suggest that biases in the recall of autobiographical memory content and the affective intensity of positive and negative memories are associated with increased transdiagnostic risk in adolescence, replicating results using the GD‐BD task in adults (Hitchcock et al., 2019), and the broader literature on the central role of these processes in the onset and maintenance of mental health problems in adults (Dalgleish & Hitchcock, 2023). Adolescence is a crucial period during which autobiographical memory is organised to support a developing self‐concept (Fivush, 2011; Habermas & Bluck, 2000; Habermas & de Silveira, 2008). Disturbances in this process may have long‐term implications for mental functioning and mental health into adulthood. Further research is necessary to explore the mechanisms through which biases in memory processing directly impact mental health and how these processes develop during adolescence. For instance, the ability to retrieve specific positive memories has been shown to support positive self‐evaluations and repair low mood in adults (Hitchcock et al., 2017; Speer & Delgado, 2017). When retrieval of positive memories is impaired, it may limit the ability to use positive autobiographical material to place boundaries around negative affect and mental content, while increased accessibility of negative content may enhance or scaffold negative biases in other domains.

In terms of implications for early intervention, our findings indicate that efforts to directly enhance category fluency for positive autobiographical memory content may be fruitful in reducing symptoms in at‐risk adolescents. Over the past decade, low‐intensity interventions that ‘train’ increased memory specificity have shown promise in reducing depression symptoms (Barry, Sze, & Raes, 2019; Neshat‐Doost et al., 2013; Pile et al., 2021). Including an explicit focus on improving recall of repeated sets of positive memories may be a useful extension or modification of these approaches. Preliminary work by Belmans, De Vuyst, Takano, and Raes (2023) has shown that a Positive Memory Specificity Training intervention was effective in reducing persistent negative memory retrieval in a community sample of adolescents aged 16–18, but further research is required to examine any long‐term impact on mental health symptoms.

Finally, we did not find support for our third hypothesis, with no differences in memory specificity between groups. Both groups recalled fewer specific memories in the bad‐day condition compared with the good‐day condition. There are several potential explanations for this. First, we recruited a community sample of adolescents and, while we split participants according to probable clinical caseness, group differences in memory specificity may only be robust in clinical samples. Several international studies using large community samples of adolescents have similarly failed to find an association between memory specificity and depression symptoms (Crane et al., 2016; Gutenbrunner, Salmon, & Jose, 2018; Lam et al., 2022). Second, it is possible that when recall is minimally constrained, as in the GD‐BD task compared with the AMT, the association between memory specificity and mental health may be reduced. While the mental life of depressed individuals is characterised by rumination on broad categorical themes (e.g. ‘I am useless’) and a tendency to overgeneralise from single negative experiences (Beck, 1976), these narratives also contain rich, highly specific memories of past events and experiences that are congruent with dominant schemas. Here, we have shown that when recall is minimally constrained, adolescents with elevated mental health symptoms are able to freely recall associated sets of specific negative memories (see also Doan et al., 2025; Warne et al., 2020). Indeed, exploratory analysis of categoric memory recall identified a trend for At‐Risk participants to recall a *lower* proportion of categoric bad‐day memories compared with Low‐Risk participants. With ongoing focus on the development of interventions that aim to directly target reduced memory specificity in adolescents (Barry, Hallford, & Takano, 2021), further research with clinical and community samples is necessary to further understand how different methods of recall may impact memory specificity and their relation to mental health.

### Limitations

This study has several potential limitations. First, our study was cross‐sectional, and we are unable to draw conclusions about the direction of effects. While there is evidence from longitudinal studies that reduced memory specificity is implicated in disorder onset in adolescence (Doan et al., 2025), there is a need for further longitudinal research in clinical and community samples to assess the predictive utility of other domains of memory processing. Second, our measure of the FAB involved retrospective recall of the affective intensity associated with past events. It is therefore possible that differences in the magnitude of the FAB between groups may have been influenced by a mood‐congruency or recency bias, although it is important to note that we did not find significant differences in the retrospective ratings of good‐day or bad‐day memories at the time at which they occurred between groups. Nonetheless, further investigation of the FAB in adolescents (and indeed adults) using more naturalistic measures (e.g. diary studies; Walker, Vogl, & Thompson, 1997) is warranted.

### Future directions

This study highlights several important areas for further research. First, there is a need for research in clinical populations and longitudinal research to further understand the role of autobiographical memory biases in the onset, maintenance, and remission of mental health problems in adolescence and through into adulthood. Second, we have shown that biases in the accessibility and affective intensity of negative and positive memories are associated with elevated risk of affective disorder in adolescence. These findings indicate potential utility in prevention approaches that seek to target biases across multiple domains of autobiographical memory recall. While interventions focusing on memory specificity have gathered traction and shown promising results (Barry et al., 2021), expanding these approaches to target multiple domains of memory recall may have a larger effect on symptoms or reducing risk than interventions targeting one domain in isolation.

## Conclusion

The present study administered a novel task, the GD‐BD task, to assess autobiographical memory processing in adolescents with elevated mental health symptoms for the first time. We demonstrated that positive biases in autobiographical memory fluency and the FAB are associated with good mental health, while adolescents at risk of affective disorder demonstrated reduced positive biases. We found no differences in memory specificity between groups. Findings extend research on autobiographical memory processing to two novel domains in adolescence and further elucidate how differences in the organisation and recall of autobiographical memory may underlie mental health, with implications for the development of memory‐based interventions in adolescents.

## Ethical considerations

All participants provided informed consent prior to taking part in the study. Ethical approval was obtained from the University of Cambridge Psychology Research Ethics Committee (PRE.2023.050; approval date May 5, 2023).

**Key points**

**What's known?**

- Distortions in multiple domains of autobiographical memory processing are implicated in the onset and maintenance of affective disorders in adults. In adolescents, research has focused on reduced memory specificity.

**What's new?**

- This study extends research on autobiographical memory processing to two novel domains – memory fluency for positive and negative events and their affective intensity.
- We found that positive biases in memory processing were associated with good mental health in adolescents, while the reduced accessibility and affective intensity of positive memories was associated with increased risk of affective disorder.

**What's relevant?**

- There has been a growing focus on interventions targeting memory specificity. Our findings suggest that augmenting these interventions to include an explicit focus on improving the accessibility of positive memories may be beneficial.

## Supporting information


**Appendix S1.** Development of the method used for scoring written autobiographical memories in the GD‐BD task.
**Table S1.** Thematic analysis of incorrectly scored memories using the automated scoring method (Mistica et al., 2024) and keywords for each theme.
**Appendix S2.** Results of main analyses with outliers included.
**Appendix S3.** Results of preregistered mixed ANCOVA to assess group differences in memory specificity.
**Appendix S4.** Supplementary analyses – continuous symptom measures.
**Table S2.** Results of ANOVAs examining memory category fluency and continuous symptom measures of depression, anxiety, and PTSD symptoms.

## Data Availability

The data that support the findings of this study are openly available in OSF at https://osf.io/r48ws.

## Endnote

1 Supplementary analyses with each symptom measure are reported in the Supporting Information File (see Appendix S4 and Table S2). These analyses showed a similar pattern of results to the main findings for continuous measures of depression, anxiety, and PTSS symptoms, respectively.
