# Supplementary material for: Remembering the bad times: Autobiographical memory fluency, specificity, and fading affect bias in adolescents at clinical risk of affective disorder
Source: J Child Psychol Psychiatry. 2026 Jun 17;67(11):1883–93. doi: 10.1111/jcpp.70191 (PMC13647094; doi:10.1111/jcpp.70191)
Supplement: Supplementary file 1 [file JCPP-67-1883-s001.docx]

**Remembering the bad times: autobiographical memory fluency, specificity and fading affect bias in adolescents at clinical risk of affective disorder**

**Supporting Information**

**Appendix S1. Development of the method used for scoring written autobiographical memories in the GD-BD Task**

Each memory description was coded for specificity using a natural language model developed for the scoring of written autobiographical memories (for further information about the model see Mistica et al., 2024).

To test the accuracy of the model for scoring GD-BD memories, we first compared the model output with 995 manually scored memories by the first-author, assumed to reflect a ‘ground truth’ (from 67 participants).^[1]^ The agreement rate was 84% (840 memories) and the disagreement rate was 11.7% (117 memories). 3.8% of the written memories (38) could not be automatically scored by the model (returning a result of ‘none’). Non-matches were inspected to identify potential inaccuracies in the model that could be rectified. Thematic analysis revealed that inaccurately scored memories tended to refer to common themes or events (e.g., referring to the death of a friend or relative, a break-up, going on holiday; see Table 1 for a summary) that were of an ambiguous duration.

To improve the accuracy of the automated scoring, a dual method of automated scoring plus manual keyword searches based on identified themes (see Table 1), and filtering based on the precision score produced by model (<0.9) was developed. This method was tested on a further 758 memories (from 59 participants) and resulted in an agreement rate of 99.1% (751 memories).

This approach was then used on the remaining 210 participants (2923 memories). First, all memories were scored by the model (Mistica et al., 2024). Each memory was then filtered depending on whether it included a relevant keyword and/or the model precision score was <0.9. These memories were then manually checked.

Following filtering (keyword searches and precision score), 1593 (54.5%) memories were manually scored (including 160 that could not be scored by the model, i.e., returned a result of ‘none’). In total, the model result differed to the manual result for 402 memories (excluding ‘none’ results).

**Table S1.** Thematic analysis of incorrectly scored memories using the automated scoring method (Mistica et al., 2024) and keywords for each theme

| Theme | Keywords | Number of memories (N = 117) |
| --- | --- | --- |
| bereavement | died, dying, pass* away, death | 22 |
| break-up | breakup, break up, broke(n) up | 11 |
| argument | argu*, fight, bullied, bully, disagreement | 10 |
| exams/exam results | exam, revis(ing), grades, results | 10 |
| holiday | holiday, flew/flight, travel, trip | 8 |
| illness/being in  hospital | hospital, illness, ill, diagnos(is/ed), cancer, rehab | 7 |
| getting a pet | cat, dog | 5 |
| getting into college | get*/got* in/into, accepted, enrol NEAR college/sixth form/uni* | 3 |
| other | - | 41 |

**Appendix S2. Results of main analyses with outliers included**

*Memory category fluency*

A repeated measures ANCOVA (co-varying for category fluency) with total number of memories recalled as the dependent variable revealed a significant main effect of condition, *F*(1,328) = 4.54, *p* = .03, pes = .01 [.00, .05], and no main effect of risk group, *F*<1. There was a significant interaction between condition and risk group, *F*(1,328) = 13.61, *p*<.001, pes = .04 [.01, .09].

*Memory specificity*

A mixed-effects logistic regression with fixed effects of condition and risk group, and random effects for participant, revealed a significant main effect of condition, such that bad-day memories were less likely to be specific, *b*= -1.0, SE = .14, z = -6.88, *p*<.001. There was no main effect of risk group, *b*= -.11, SE = .20, *z* = -.56, *p*=.58, or interaction between risk group and condition, *b*= -.11, SE=.18, *z* = -.61, *p*=.54.

*Fading Affect Bias (FAB)*

One-sample t tests with a reference value of zero demonstrated a significant FAB for both groups: Low Risk, M = .93, SD = .88, *t*(126) = 11.93, *p*<.001, *d* = 1.06 [.84, 1.27]; At Risk, M = .65, SD = .91, *t*(206) = 10.30, *p*<.001, *d* = .72 [.56, .87]. A one-way ANOVA with the FAB index as the dependent variable revealed that the FAB index was significantly larger in the Low Risk group compared to the At Risk group, *F*(1,332) = 7.55, *p* = .006, eta = .02 [.00, .06].

**Appendix S3. Results of preregistered mixed ANCOVA to assess group differences in memory specificity**

A Group X Condition repeated measures ANCOVA, with the proportion of specific memories recalled as the dependent variable, revealed a main effect of condition, *F*(1,316) = 88.23, *p*<.001, $n_{p}^{2}$ = .22 [.14, .29], such that good-day memories were more specific than bad-day memories. There was no main effect of risk group, *F*(1,316) = 2.66, *p* = .10, $n_{p}^{2}$ = .01 [.00, .04], or interaction between risk group and condition, *F*(1,316) = 1.06, *p* = .30, $n_{p}^{2}$= .01 [.00, .03].

**Appendix S4. Supplementary analyses – continuous symptom measures**

*Memory category fluency*

Results of repeated measures ANOVAs predicting the total number of memories recalled from symptoms (depression, anxiety and PTSD symptoms, respectively) and condition (good-day vs bad-day) and their interaction are reported in Table 1.

**Table S2.** Results of ANOVAs examining memory category fluency and continuous symptom measures of depression, anxiety and PTSD symptoms

| Effect | df | MSE | F | pes | *p* |
| --- | --- | --- | --- | --- | --- |
| Model 1: Depression symptoms | | | | | |
| Depression symptoms | 1, 316 | 10.80 | 2.34 | .007 | .127 |
| Condition | 1, 316 | 3.14 | 79.32 *** | .201 | <.001 |
| Depression: Condition | 1, 316 | 3.14 | 33.03 *** | .095 | <.001 |
| Model 2: Anxiety symptoms | | | | | |
| Anxiety symptoms | 1, 316 | 10.81 | 2.00 | .006 | .158 |
| Condition | 1, 316 | 3.40 | 18.32 *** | .055 | <.001 |
| Anxiety:Condition | 1, 316 | 3.40 | 5.95 * | .018 | .015 |
| Model 3: PTSD symptoms | | | | | |
| PTSD symptoms | 1, 316 | 10.84 | 0.94 | .003 | .333 |
| Condition | 1, 316 | 3.26 | 63.86 *** | .168 | <.001 |
| PTSD:Condition | 1, 316 | 3.26 | 20.54 *** | .061 | <.001 |

** p* <.05, *** <.001.

*Memory specificity*

We conducted three generalised linear mixed effects models to examine the association between memory specificity and depression, anxiety and PTSD symptoms, respectively. Models included fixed effects of condition and symptoms, their interaction, and random effects for participant.

There was no main effect of depression symptoms (*b*=-.01, SE = .009, z=1.11, *p*=.27), or interaction between depression symptoms and condition (*b*=0.004, SE = .008, z=.48, *p*=.63). As with the main results, there was a significant effect of condition, such that bad-day memories were less likely to be specific (*b*=-1.01, SE=.21, z=4.79, *p*<.001).

There was no main effect of anxiety symptoms (*b*=.02, SE=.09, z=.20, *p*=.84), or interaction (*b*=-.13, SE=.09, z=1.50, *p*=.14). There was a significant main effect of condition, such that bad-day memories were less likely to be specific (*b*=-1.10, SE=.09, z=12.22, *p*<.001).

There was no main effect of PTSD symptoms (*b*=-.004, SE=.01, z=.51, *p*=.61), or interaction (*b*=-.01, SE=.01, z=.99, *p*=.32). There was a significant main effect of condition, such that bad-day memories were less likely to be specific (*b*=-.93, SE=.19, z=4.86, *p*<.001).

*Fading Affect Bias (FAB)*

Linear regressions revealed a significant effect of depression (*F*(1,316)=21.8, *p*<.001, R^2^=.06, *b*=-.02) and PTSD symptoms (*F*(1,316)=9.54, *p*=.002, R^2^=.03, *b*=-.01) on the FAB index. Anxiety symptoms were not a significant predictor of FAB (*F*(1,316)=3.58, *p*=.059, R^2^=.01, *b*=-.10).

1. Inter-rater agreement on memories rated independently by the first and last author (n=995) indicated good reliability for manually-scored memories, κ = .91.
